# Coordinated MBW, GIS, and RSL Regulatory Networks in Plant Epidermal Patterning Under Environmental Cues

**DOI:** 10.3390/ijms27156824

**Published:** 2026-07-30

**Authors:** Muhammad Umair Yasin, Zulqarnain Haider, Irshan Ahmad, Yinbo Gan

**Affiliations:** 1 Zhejiang Key Laboratory of Crop Germplasm, Department of Agronomy, College of Agriculture and Biotechnology, Zhejiang University, Hangzhou 310058, China; umairyasin@zju.edu.cn (M.U.Y.); 12216159@zju.edu.cn (Z.H.);; 2 Institute of Urban Agriculture, Chinese Academy of Agricultural Sciences, Chengdu Agricultural Science and Technology Center, Chengdu 610299, China

**Keywords:** epidermal patterning, trichomes, root hairs, MBW regulatory network, GIS proteins, RSL transcription factors, abiotic stress, phytohormone signaling, adaptive trade-offs

## Abstract

The plant epidermis, adorned with trichomes and root hairs, represents a critical interface where developmental programming and environmental responses converge. Although the genetic basis of epidermal patterning has been extensively characterized in model systems, how these pathways are modulated under abiotic stress remains incompletely understood. This review integrates recent advances in epidermal development and stress biology, focusing on MYB–bHLH–WD40 (MBW) complexes, GIS-family C2H2 zinc-finger proteins, and ROOT HAIR DEFECTIVE SIX-LIKE (RSL) transcription factors. These regulators participate in interconnected, organ-specific networks that coordinate trichome and root-hair development. Their activities are shaped by gibberellin–brassinosteroid interactions, ethylene–auxin coordination, jasmonate and abscisic acid signaling, and cytokinin- and nutrient-responsive pathways. We further discuss how reactive oxygen species and calcium oscillations translate transcriptional regulation into polarized cell growth. The resulting epidermal plasticity reflects trade-offs among growth, defense, resource acquisition, and conservation. By integrating single-cell transcriptomics, nutrient sensing, and evolutionary perspectives, this review provides a framework for understanding environmentally responsive epidermal development and identifies opportunities for improving crop resilience. The resulting framework identifies testable opportunities for crop improvement, while emphasizing that native network equivalence, pleiotropic effects, and field-level stress benefits remain to be established in crop species.

## 1. Introduction

The plant epidermis constitutes a critical interface between the organism and its environment, functioning as the first line of defense against abiotic stresses such as drought, salinity, and temperature extremes [1]. Specialized epidermal structures, including trichomes and root hairs, play essential roles in plant adaptation. Trichomes reduce water loss, reflect excess radiation, and provide protection against herbivores and pathogens [1,2], whereas root hairs enhance water and nutrient uptake and facilitate interactions with the soil microbiome [3]. The development of these structures is highly plastic, enabling dynamic adjustments in response to environmental cues [4,5].

This plasticity is governed by integrated genetic and hormonal networks. Key transcription factors, including C2H2 zinc-finger proteins (e.g., GIS family), HD-Zip IV, and MYB proteins, regulate epidermal cell fate and differentiation by integrating developmental and environmental signals [6,7,8]. Hormonal pathways act as central modulators of these networks. Auxin and its polar transport system are crucial for epidermal patterning, while ethylene promotes adaptive root traits under stress [9,10]. Additional hormones, including abscisic acid, jasmonates, and brassinosteroids, further refine developmental outputs through context-dependent regulatory coupling [2,11].

Recent advances in single-cell and spatial transcriptomics have revealed cell-type-specific stress responses, demonstrating that plant resilience arises from coordinated yet distinct cellular programs [5,12,13]. Together with classical genetic and physiological studies, these approaches are clarifying how developmental programs are adjusted under changing environmental conditions. Such plasticity also involves trade-offs: while trichomes and root hairs can enhance stress tolerance and resource acquisition, their formation incurs metabolic costs that may affect growth and productivity [3,14,15]. This review synthesizes current knowledge on the genetic and hormonal regulation of epidermal development under abiotic stress, emphasizing signaling integration and the adaptive trade-offs that shape plant resilience.

## 2. Core Transcriptional Networks for Epidermal Patterning

### 2.1. Core Regulatory Mechanisms of Trichome Initiation and Morphogenesis

Trichome initiation and morphogenesis are governed in *Arabidopsis thaliana* by an MYB–bHLH–WD40 (MBW) activator complex composed of the R2R3-MYB GL1, the bHLH proteins GL3/EGL3, and the WD40 protein TTG1. This complex promotes trichome fate and patterning [16,17]. Spatial distribution is generated through lateral inhibition: the activating complex induces mobile R3-MYB inhibitors such as CAPRICE (CPC) and TRIPTYCHON (TRY), which compete for bHLH binding and suppress trichome fate in neighboring cells [16,18]. Although MBW regulatory assemblies are broadly conserved across plant species, variation in their molecular composition and downstream targets has contributed to the diversification of epidermal patterning.

Species-specific modifications of this regulatory architecture generate diverse trichome types. In tomato, antagonistic MYB factors SlMYB72 and SlMYB75 form competing MBW complexes to fine-tune trichome density [19], whereas MYB-like repressors (GCR1/2) regulate glandular trichome formation through developmental timing pathways [20]. Functional conservation is further supported by cross-species complementation, such as PaTTG1 restoring trichome formation in *Arabidopsis* [21], and by candidate bHLH regulators identified in tea plants [22].

**Figure 1:**
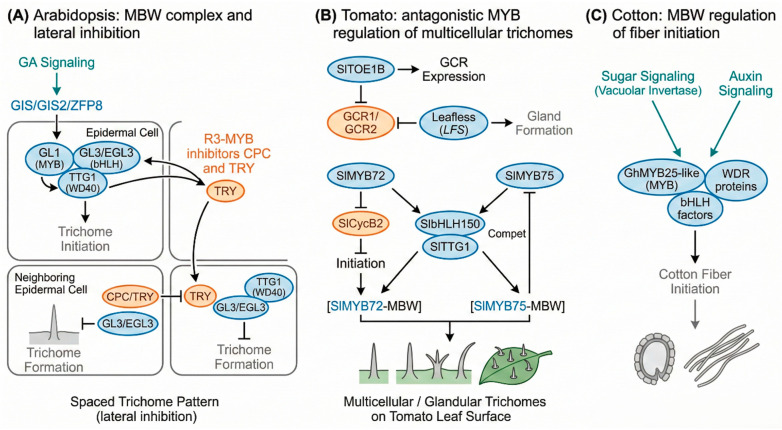
Genetic regulatory networks controlling trichome initiation and morphogenesis in *Arabidopsis* (**A**), tomato (**B**), and cotton (**C**).

Trichome development is further modulated by epigenetic and post-transcriptional mechanisms. Histone methylation mediated by SDG26 represses key regulators, while miRNAs (e.g., miR6155) influence trichome density through transcription factor targeting [23,24]. These multi-layered controls ensure precise spatiotemporal regulation [25,26]. Hormonal integration is mediated by C2H2 zinc-finger proteins such as GIS, GIS2, and ZFP8, which link gibberellin signaling to MBW activity and developmental timing pathways [17]. Natural variation in MBW components further drives morphological diversity [27]. In cotton, MBW-regulated fiber initiation is additionally coupled to sugar signaling, highlighting metabolic integration [25]. Together, these studies underscore the conserved yet adaptable nature of the MBW-centered network in shaping trichome development across species (Figure 1; Table 1).

### 2.2. The GIS Clade: Hormonal and Developmental Integrators of Trichome Development

Trichome development is strongly influenced by hormonal signaling, with a central role played by the C2H2 zinc-finger transcription factor clade represented by GLABROUS INFLORESCENCE STEMS (GIS). GIS acts downstream of gibberellin (GA) signaling and upstream of the core regulator GLABROUS1 (GL1), mediating GA-induced trichome initiation in inflorescence organs [33]. In addition to initiation, GIS also regulates trichome morphogenesis by repressing branching through a pathway downstream of STICHEL (STI) [34].

The GIS family includes GIS2, ZFP5, ZFP6, ZFP8, and GIS3, forming a regulatory network that integrates hormonal cues to fine-tune trichome development [30,31]. Although these proteins share functional similarities, they exhibit specialization in hormonal responsiveness and spatial activity. GIS primarily mediates GA-dependent trichome initiation in stems, whereas GIS2 and ZFP6 integrate both GA and cytokinin signals, particularly in floral organs [30,35,36]. A hierarchical structure exists within this clade: GIS3 acts upstream by directly regulating GIS and GIS2, while ZFP5 controls trichome initiation through activation of ZFP8 [31,37,38]. These interactions channel hormonal signals toward activation of the core MBW complex.

The GIS pathway is further integrated with developmental timing and patterning networks. GIS directly regulates SPL15, linking GA signaling with the miR156/SPL module [39], while interactions with the miR172–TOE1/TOE2 pathway connect trichome development with phase transitions [17]. Genetic evidence also places GIS downstream of R3 MYB repressors such as TCL1 [40].

**Figure 2:**
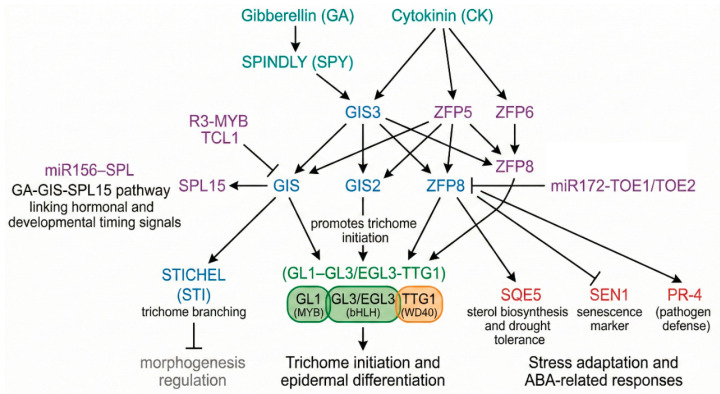
GIS-centered regulatory network integrating hormonal, developmental, and stress-associated pathways in the *Arabidopsis* shoot epidermis.

Beyond developmental regulation, GIS2 and ZFP8 have been associated with drought- and defense-related gene expression and with ABA-responsive transcriptional programs [36]. These findings extend the GIS regulatory network beyond trichome initiation and suggest links among epidermal development, hormone signaling, and environmental response. Defining how these associations influence trichome plasticity across tissues and environmental conditions remains an important direction for future research (Figure 2).

### 2.3. Genetic Control of Root-Hair Cell Fate and Elongation

**Figure 3:**
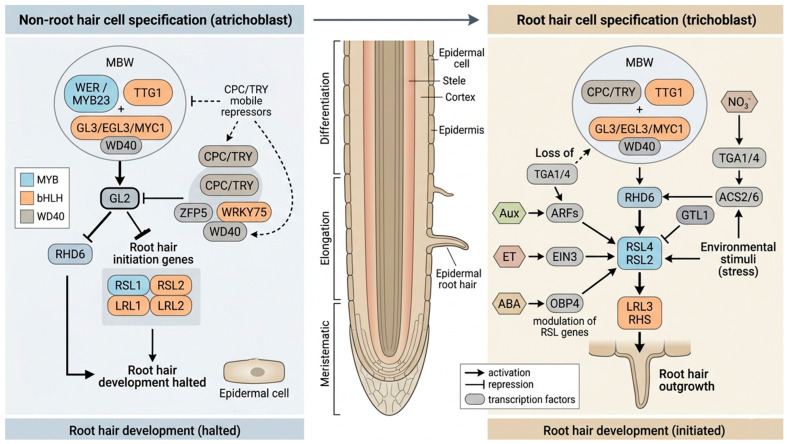
Root epidermal cell-fate specification and root-hair growth. The activation of the WER–GL3/EGL3–TTG1 MBW complex promotes GL2 and non-hair fate; CPC/TRY/ETC1 inhibit MBW activity and permit RHD6/RSL-dependent hair development. Solid lines denote established regulatory directions, whereas dashed lines denote context-dependent convergence.

Root-hair development combines position-dependent cell-fate specification with polarized tip growth. In *Arabidopsis thaliana*, epidermal cells over the cleft between two cortical cells (H positions) can become trichoblasts, whereas cells at N positions generally adopt non-hair identity [41]. The WER–GL3/EGL3–TTG1 activation of the MBW complex promotes non-hair fate by activating GL2 [42]. Mobile R3-MYB proteins, including CPC and TRY, inhibit this complex in neighboring cells, thereby permitting RHD6/RSL-dependent hair-cell differentiation [41] (Figure 3).

Root-hair initiation and elongation are controlled by bHLH transcription factors, with RHD6 and RSL1 initiating hair formation and RSL4 acting as a central regulator of elongation [43,44,45]. RSL4 directly activates genes involved in vesicle trafficking, cytoskeleton organization, and cell wall remodeling [46]. Its activity is tightly regulated: ZFP1 represses RHD6/RSL genes in non-hair cells [47], while GTL1/DF1 form a feedback module that limits hair growth by repressing RSL4 [48]. Comparable regulatory strategies are observed across land plants, despite divergence in specific repressors [49].

Hormonal and environmental signals converge on the root-hair pathway through multiple molecular routes. Auxin promotes RSL expression through ARF-dependent regulation [50]. Ethylene activates RSL4 through EIN3/EIL1 cooperation with RHD6/RSL1 [43], and jasmonate stimulates elongation by relieving JAZ-mediated repression of RHD6/RSL1 [51]. In contrast, ABA-activated ABF proteins suppress RHD6 during salinity stress [52]. Together, these inputs adjust root-hair initiation and elongation according to developmental and environmental conditions.

### 2.4. Shared Regulatory Logic and Evolutionary Parallels

Shoot and root epidermal patterning reuse MBW components and mobile R3-MYB inhibitors, but they generate distinct developmental outputs. The GL1-based shoot MBW promotes trichome fate, whereas the WER-based root MBW activates GL2 and promotes non-hair identity; inhibition of the root MBW permits the RHD6/RSL hair pathway [16,18,28]. This reuse of MBW components and mobile inhibitors constitutes a conserved activator–inhibitor architecture adapted to organ-specific developmental programs.

Functional conservation is supported by cross-species complementation, in which homologous regulators restore epidermal defects in *Arabidopsis* [29,41,53]. Similar MBW-centered assemblies occur across diverse species, but differences in component composition, regulatory interactions, and downstream targets have contributed to evolutionary diversification [54]. For example, mutations in *GL1*, *TCL1*, and *TRY* enabled trichome formation in novel tissues [55], whereas loss of WER-like genes produces random root-hair patterning in some species [41,56].

Ethylene can modify root epidermal patterning by interfering with WER-based MBW activity through EIN3/EIL1–TTG1 interactions and by RSL4-dependent feedback on GL2 [57]. The resulting regulatory flexibility provides opportunities for tissue-specific modification of epidermal traits and underscores the importance of considering the distinct mechanisms operating in shoot and root tissues [58,59]. The major hormonal and environmental regulators of epidermal cell fate and root-hair development are summarized in Table 2.

### 2.5. Translation to Cereal and Legume Crops: Conserved Components and Emerging Opportunities

Rice RSL4 orthologs restore root-hair growth in an *Arabidopsis rsl4* mutant and bind conserved ROOT HAIR SPECIFIC promoter elements [64]. Expression of *TaRSL2* homoeologs correlates with root-hair length across wheat ploidy levels, and *TaRSL2*-*4D* overexpression increases root-hair length in *Arabidopsis* [65]. In the legume *Lotus japonicus*, LjRHL1 is required for root-hair development and for root-hair-dependent rhizobial infection [66]. Together, these studies demonstrate that core downstream functions of RSL-related pathways are retained across species. Their contributions to environmentally induced root-hair plasticity in crops, however, remain less well-characterized.

Rice OsTCL1 interacts with *Arabidopsis* GL3 and changes *Arabidopsis* trichome and root-hair phenotypes, yet comparable overexpression does not reproduce those effects in rice [67], illustrating that biochemical compatibility in a heterologous system does not necessarily predict native network activity. Cereal root hairs are promising breeding targets [68], and wheat and maize studies document stress-responsive root phenotypes [11,69]. Elucidating how native MBW-, GIS-, and RSL-related pathways shape these traits will be essential for translating epidermal biology into crop improvement.

## 3. Phytohormonal Co-Regulation of Epidermal Development Under Stress

### 3.1. Jasmonate Signaling

**Figure 4:**
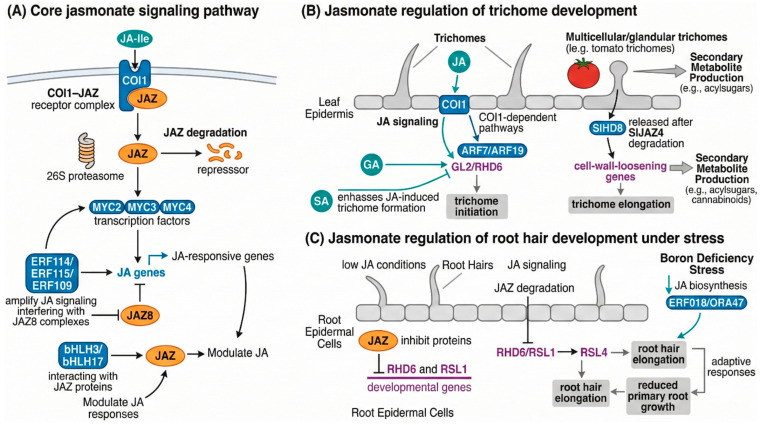
Jasmonate signaling regulates epidermal development under stress. (**A**) JA-Ile perception by the COI1–JAZ complex releases MYC transcription factors. (**B**) JA promotes trichome initiation, elongation, and secondary metabolism. (**C**) JA relieves JAZ-mediated repression of RHD6/RSL1 and promotes RSL4-dependent root-hair elongation.

Jasmonate (JA) is a central regulator linking environmental stress to epidermal development. Through the conserved COI1–JAZ co-receptor system, JA modulates both trichome and root-hair formation, thereby adjusting plant–environment interactions in a context-dependent manner (Figure 4). JA signaling is initiated by perception of jasmonoyl-L-isoleucine (JA-Ile) by the COI1–JAZ complex, leading to JAZ degradation and activation of transcription factors such as MYC2/3/4 [70,71]. Additional regulators, including bHLH IIId proteins and ERF114/115/109, modulate this pathway through feedback interactions with JAZ proteins, refining JA responses [71,72].

JA positively regulates trichome development. In *Arabidopsis*, JA-induced trichome initiation requires COI1, ARF7/ARF19, and core regulators such as GL2 and RHD6 [73,74], and is further shaped by hormone-dependent modulation, including combined promotion with gibberellin treatments and opposing effects of salicylic acid in the experiments cited in [74]. In multicellular and glandular trichomes, JA promotes elongation and secondary metabolite production. For example, JA releases SlHD8 from JAZ repression in tomato, activating genes involved in cell wall remodeling, while enhancing synthesis of defense compounds such as acylsugars and cannabinoids, often at a cost to growth [75,76,77].

JA also promotes root-hair elongation by targeting the RHD6–RSL pathway. JAZ proteins repress RHD6/RSL1 under basal conditions, whereas JA-induced degradation activates RSL4-dependent growth [51,71]. Under abiotic stress, such as boron deficiency, JA biosynthesis is induced, leading to reduced primary root growth but enhanced root-hair development, improving nutrient acquisition [71,78].

### 3.2. Brassinosteroid–Gibberellin Co-Regulation

**Figure 5:**
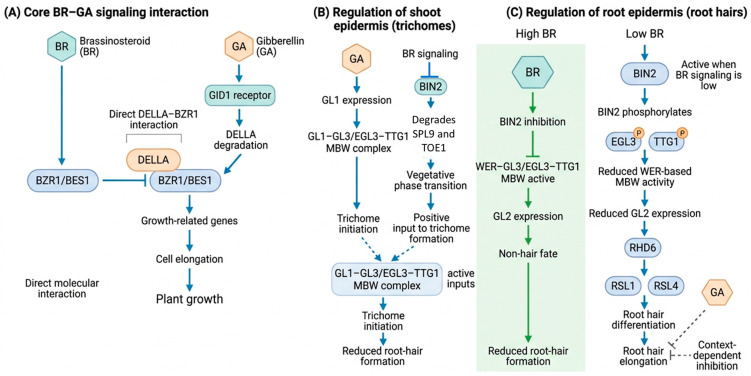
Brassinosteroid–gibberellin co-regulation of epidermal development. (**A**) BR inhibits BIN2 through BRI1 signaling, while GA promotes DELLA degradation and releases BZR1/BES1 activity. (**B**) GA- and BR-associated pathways provide parallel positive inputs to shoot trichome development. (**C**) High BR suppresses BIN2 and favors WER-MBW–GL2-dependent non-hair fate; low BR permits active BIN2 to phosphorylate EGL3/TTG1, inhibit WER-MBW activity, and favor hair-cell differentiation.

Brassinosteroids (BR) and gibberellins (GA) regulate plant growth through partially connected pathways. Their crosstalk is anchored by interactions between GA-regulated DELLA repressors and BR-activated BZR1/BES1 proteins, while epidermal responses also reflect tissue-specific inputs from each pathway (Figure 5).

At the molecular level, DELLAs directly bind BZR1 and inhibit its DNA-binding activity, whereas GA promotes DELLA degradation and releases BZR1/BES1-dependent transcription [79,80,81]. The DELLA–BZR1/BES1 interface therefore links GA and BR signaling to the regulation of growth-related genes.

The effects of BR and GA on epidermal development are context dependent. In shoots, GA promotes *GL1* expression, whereas BR signaling influences vegetative phase change through BIN2-dependent control of SPL9 and TOE1 stability; these pathways provide parallel positive inputs to trichome production [82,83]. In roots, enhanced BR signaling inhibits root-hair formation because BR suppresses BIN2 activity. When BR signaling is low or impaired, active BIN2 phosphorylates EGL3 and TTG1, reduces WER-based MBW activity and GL2 expression, and thereby favors hair-cell differentiation [60]. Thus, BIN2 activity—not BR activation of BIN2—inhibits the root MBW complex. GA can also reduce root-hair elongation in specific contexts [84].

This signaling connection operates within a broader regulatory network involving PHYTOCHROME-INTERACTING FACTORS (PIFs), forming a DELLA–BZR1–PIF module that integrates light and hormonal signals to control growth-related genes [81,85]. Such integration enables flexible coordination of developmental processes, exemplified during seed germination where BR and GA jointly regulate cell elongation via shared targets like GASA6 [85].

### 3.3. Ethylene and Abscisic Acid

**Figure 6:**
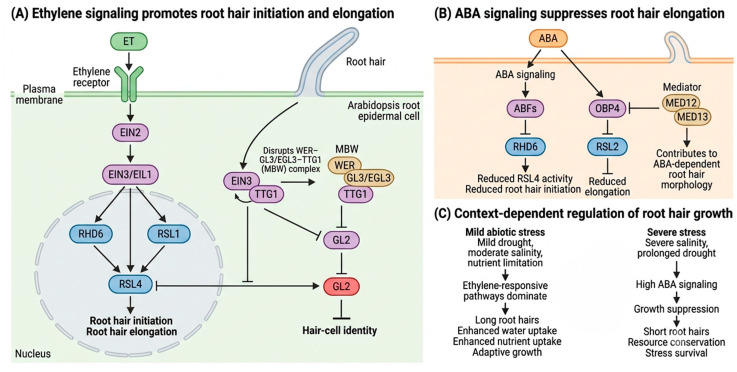
Ethylene and ABA regulate root-hair development under stress. (**A**) Ethylene promotes initiation and elongation through EIN3/EIL1–RHD6/RSL1 cooperation and RSL4 activation. (**B**) ABA suppresses growth through OBP4-mediated RSL2 repression and ABF-mediated inhibition of RHD6. (**C**) Their context-dependent balance coordinates resource acquisition and growth restraint across stress intensities.

Ethylene and abscisic acid (ABA) are key regulators of plant responses to abiotic stress and often exert contrasting effects on epidermal development. Their context-dependent influences on core regulatory networks enable dynamic adjustment of root-hair growth under environmental stress (Figure 6). Ethylene is a strong positive regulator of root-hair development. The transcription factors EIN3/EIL1 interact directly with RHD6 and RSL1 to co-activate RSL4, thereby promoting root-hair initiation and elongation [43]. Ethylene also influences epidermal cell fate by repressing GLABRA2 (GL2), the non-hair cell determinant. This occurs through EIN3 interaction with TTG1, which disrupts the MBW complex, together with RSL4-dependent feedback on GL2 [57]. These mechanisms allow ethylene to override positional patterning and enhance root-hair formation under stress [86].

In contrast, ABA generally suppresses root-hair growth, particularly under severe stress. ABA induces the transcription factor OBP4, which represses RSL2 expression and inhibits elongation [63]. Under salt stress, ABA-activated ABF transcription factors directly interact with RHD6, reducing its activity and downstream gene expression [52]. Additional components, such as Mediator subunits MED12/13, contribute to ABA-dependent control of root-hair morphology [87].

The balance between ethylene- and ABA-regulated pathways depends on stress intensity and developmental context. Under mild stress, ethylene-associated growth responses may support longer root hairs and enhanced resource acquisition, whereas under severe or prolonged stress, ABA signaling can predominate and suppress growth to conserve resources [84,86,88]. Given the central role of root hairs in water and nutrient uptake, this hormonal balance has important implications for plant resilience and productivity [89,90].

### 3.4. Cytokinins

**Figure 7:**
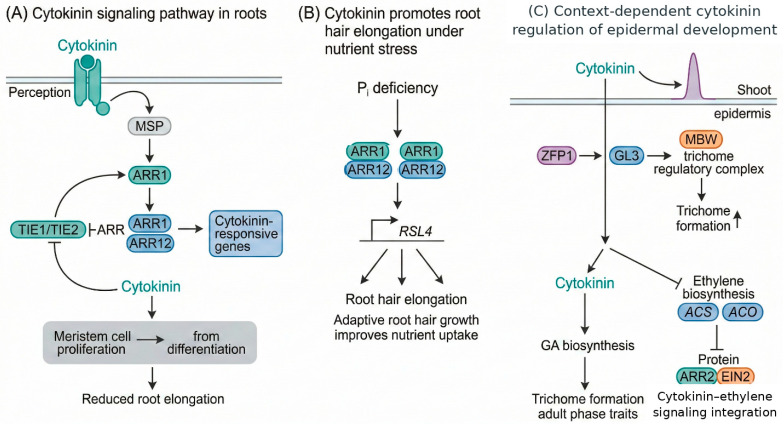
Cytokinin signaling regulates epidermal development. (**A**) Cytokinin activates type-B ARRs through the multistep phosphorelay pathway, with TIE proteins providing negative feedback. (**B**) ARR1/ARR12 directly activate RSL4 to promote root-hair elongation under phosphate deficiency. (**C**) Cytokinin influences trichome regulation, GA biosynthesis, and spatially specific ethylene production through tissue- and stage-dependent pathways.

Cytokinins (CKs) play context-dependent roles in epidermal development, acting as modulators of cell proliferation and differentiation through extensive regulatory coupling with other hormonal pathways (Figure 7). CK signaling is mediated via a multistep phosphorelay system that activates type-B *ARABIDOPSIS* RESPONSE REGULATORS (ARRs), which regulate downstream gene expression [91]. A key feature of CK action is its spatiotemporal specificity, with distinct effects across root developmental zones [92]. CK generally inhibits root elongation by promoting the transition from proliferation to differentiation, partly through modulation of auxin responses and cell wall properties [93]. This activity is tightly controlled by feedback loops, such as ARR1-mediated activation of TIE repressors, which subsequently attenuate CK signaling [94].

Despite its inhibitory role in primary-root growth, CK can promote root-hair elongation under nutrient stress. During phosphate deficiency, ARR1 and ARR12 directly activate RSL4, linking CK signaling to the core root-hair developmental machinery [62]. CK also acts alongside auxin and ethylene to influence root-hair development [84]. In the shoot epidermis, CK promotes trichome initiation through factors such as ZFP1, which acts upstream of GL3 [95,96], and contributes to phase-dependent trichome production in coordination with gibberellin signaling [97]. Type-B ARRs regulate genes in auxin, ethylene, and ABA pathways and can interact with components such as EIN2 [91,97]. These connections allow CK to fine-tune epidermal patterning according to tissue identity, developmental stage, and nutrient status.

### 3.5. Gibberellins and DELLA Repressors

**Figure 8:**
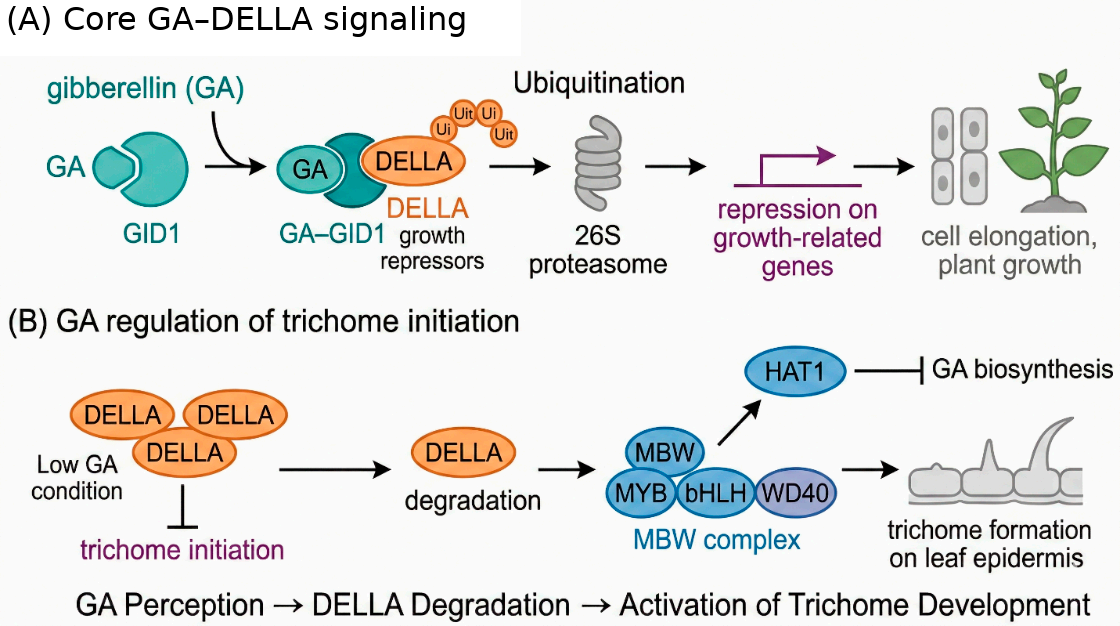
GA–DELLA signaling regulates trichome development and growth. (**A**) GA perception by GID1 triggers DELLA degradation and releases growth-related transcription factors. (**B**) GA-induced DELLA degradation permits activation of trichome-regulatory genes upstream of the MBW complex.

Gibberellins (GAs) are key regulators of plant growth whose signaling is mediated through DELLA proteins, central repressors that integrate hormonal and environmental signals. The GA–DELLA module functions as a major signaling hub influencing epidermal development by modulating core genetic networks for trichome and root-hair formation (Figure 8). GA signaling promotes growth through DELLA degradation. Binding of bioactive GA to its receptor GID1 triggers ubiquitination and proteasomal degradation of DELLA proteins, thereby releasing repression on downstream transcriptional programs [98]. Although DELLAs lack DNA-binding capacity, they regulate gene expression via interactions with multiple transcription factors, integrating signals from auxin, brassinosteroids, jasmonates, and light pathways [99].

In trichome development, DELLA proteins act as negative regulators, and their GA-induced degradation is required for activation of trichome initiation pathways. This regulation occurs upstream of the MBW complex, with GA promoting the expression of key trichome regulators [100,101]. Feedback mechanisms further refine this process; for example, the HD-ZIP II factor HAT1 modulates GA homeostasis through interaction with DELLA proteins [102]. Additional regulatory coupling with developmental regulators, such as the miR319a/TCP module, links GA signaling to trichome density and defense responses [32].

GA–DELLA signaling is also tightly connected to developmental phase transitions. Through interactions with SPL and PIF transcription factors, DELLAs coordinate vegetative phase change, linking trichome formation with broader developmental processes [99,103]. Regulatory connections with other hormones, including cytokinins, further highlight the integrative role of this pathway in balancing growth and environmental responses [97] (see Table 3).

## 4. Systemic Signal Hubs in Stress-Regulated Epidermal Development

### 4.1. Reactive Oxygen Species and Calcium Signaling

Polarized root-hair growth depends on tightly coordinated cellular processes regulated by reactive oxygen species (ROS) and calcium (Ca^2+^), which function as interconnected secondary messengers controlling cell wall dynamics, vesicle trafficking, and cytoskeletal organization. Root-hair elongation is driven by an oscillatory ROS–Ca^2+^ circuit. Tip-focused Ca^2+^ gradients coincide with oscillatory ROS production, primarily generated by the NADPH oxidase RBOHC/RHD2 [109,110,111]. ROS activate Ca^2+^ channels such as CNGCs, promoting Ca^2+^ influx, which in turn enhances RBOHC activity, forming a positive feedback loop essential for sustained tip growth [111,112]. This circuit is spatially controlled by ROP GTPases, with active ROP2 directly stimulating ROS production and polarity establishment [109].

**Figure 9:**
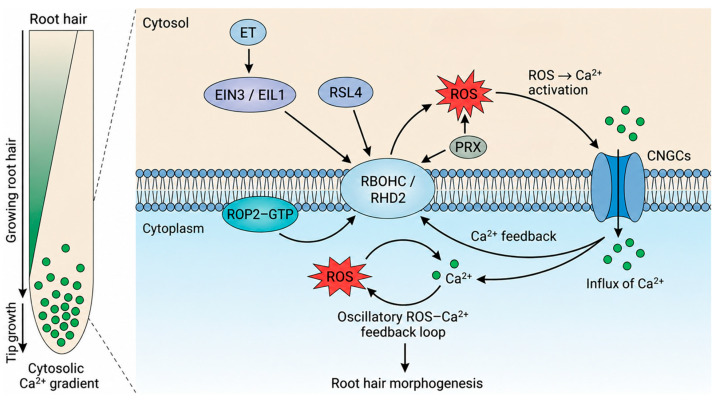
Root-hair morphogenesis is regulated by an oscillatory ROS–Ca^2+^ feedback loop. RBOHC/RHD2-derived apoplastic ROS activates Ca^2+^ influx through CNGCs, while the tip-focused cytosolic Ca^2+^ signal reinforces RBOHC/RHD2 activity to sustain polarized growth.

The ROS–Ca^2+^ module is closely integrated with hormonal and transcriptional networks. Ethylene signaling promotes root-hair initiation by upregulating ROS-producing enzymes such as RBOHC and PRX44 via EIN3/EIL1 [106]. Environmental stimuli, including cadmium stress, similarly enhance ROS levels and modulate expression of cell wall-related genes such as EXPANSINs [107]. Central to this integration is RSL4, which directly activates ROS-generating enzymes, linking developmental regulation to growth execution [111]. Precise spatial and temporal control of ROS is critical. Developmental gradients of superoxide and hydrogen peroxide are regulated by factors such as UPBEAT1, coordinating cell proliferation and differentiation [113]. ROS homeostasis is further maintained by antioxidants such as flavonols; disruption of this balance alters ROS distribution and increases root-hair formation [114]. Together, the ROS–Ca^2+^ network translates regulatory signals into polarized cell growth (Figure 9; Table 4).

### 4.2. Nutrient-Sensing Pathways as Paradigms of Systemic Coordination

**Figure 10:**
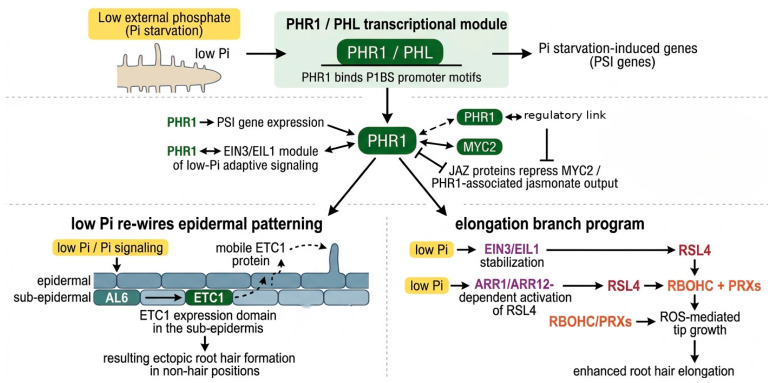
Phosphate starvation reprograms root-hair patterning and elongation through a PHR1-centered transcriptional network. Arrowheads indicate activation or promotion, blunt-ended lines indicate repression or inhibition, and dashed lines indicate indirect, proposed, or context-dependent relationships. Colors distinguish regulatory components or signaling pathways.

Plant responses to nutrient deficiency exemplify how systemic signals coordinate local epidermal development. The phosphate (Pi) starvation response is particularly well characterized, revealing a regulatory network that reprograms root-hair development to enhance nutrient acquisition (Table 5; Figure 10). Central to this response is the MYB-CC transcription factor PHOSPHATE STARVATION RESPONSE 1 (PHR1), which, together with PHL homologs, regulates a broad set of Pi starvation-induced genes by binding P1BS promoter elements [115,116,117]. PHR1 also integrates systemic growth responses; under Pi deficiency, a PHR1–EIN3/EIL1 module reallocates resources, suppressing root and cotyledon growth while promoting hypocotyl elongation [118].

Pi starvation strongly enhances root-hair density and length through multiple pathways. One mechanism involves ALFIN-LIKE 6 (AL6), which regulates genes including the mobile R3 MYB factor ETC1. Induction of ETC1 expression in sub-epidermal tissues allows its protein to override positional cues, promoting ectopic root-hair formation [61,119]. In parallel, Pi deficiency promotes root-hair elongation via upregulation of RSL4, mediated by stabilization of EIN3/EIL1 and cytokinin-dependent activation through ARR1/ARR12 [62,120].

Regulatory coupling further integrates Pi signaling with hormonal and defense pathways. PHR1 interacts with MYC2 to co-regulate jasmonate-responsive genes, with JAZ proteins modulating this interaction [108]. Additionally, beneficial microbes can enhance Pi responses by modulating host hormone signaling and root architecture [121]. Importantly, the contribution of root hairs to nutrient uptake is nutrient-specific. While critical for phosphate acquisition, their role in sulfate uptake is limited, with overall root biomass playing a larger role [122].

**Table 5:** Nutrient sensing and systemic coordination of epidermal development.

Nutrient/Pathway	Central Regulator	Mechanism of Action on Epidermis	Hormonal Integration	Key Target Genes in the Epidermal Network	References
Phosphate (Pi) Starvation	PHR1/PHR1-like (PHL) TFs	Binds P1BS motif; acts as central transcriptional hub for Pi Starvation Response (PSR).	Physically interacts with MYC2 to enhance JA signaling. Promotes ethylene/auxin accumulation.	Directly/indirectly upregulates RSL4, RBOHC, ETC1.	[108,115,116]
	ALFIN-LIKE 6 (AL6)	Required for root-hair elongation under low Pi.	Links Pi starvation and light signaling.	Positively regulates ETC1 transcript stability.	[119]
	ETC1 (R3 MYB)	Pi starvation induces its expression in the sub-epidermis.	Provides a mobile pro-hair signal that overrides positional patterning.	Promotes root-hair initiation in N-position cells.	[61]
Sulfur Deficiency	SULTR1;1, SULTR1;2	High-affinity sulfate transporters; uptake rate not directly correlated with root-hair density.	S-deficiency increases root-hair number, but mechanism distinct from Pi.	Uptake is influenced more by root biomass than hair-specific regulation.	[122]

### 4.3. Stress-Responsive Epidermal Plasticity Across Species

Stress-responsive epidermal development has been examined in diverse tissues and species (Table 6). Salinity inhibits *Arabidopsis* root-hair elongation through ABA-activated ABFs that interact with RHD6 [52], whereas phosphate deficiency promotes ectopic hair formation and elongation through ETC1-, EIN3/EIL1-, and ARR1/ARR12–RSL4-dependent pathways [61,62]. Cadmium stimulates ROS accumulation and root-hair elongation [107], while drought, water limitation, cold, and combined drought–salinity treatments alter epidermal or root phenotypes in *Arabidopsis*, Shepherdia, maize, and wheat [9,11,15,69,123].

Across these studies, stress-responsive changes in trichome or root-hair traits are clear, but the underlying regulators and physiological consequences are resolved to different degrees. Mechanistic insight is strongest in *Arabidopsis* nutrient and salinity responses, whereas many crop studies primarily describe phenotype- and transcriptome-level associations. Expanding functional analyses in cereals, legumes, and other crops will clarify how conserved regulatory pathways contribute to stress adaptation and growth–defense allocation.

## 5. An Integrated Framework for the Epidermal Stress Response and Adaptive Trade-Offs

**Figure 11:**
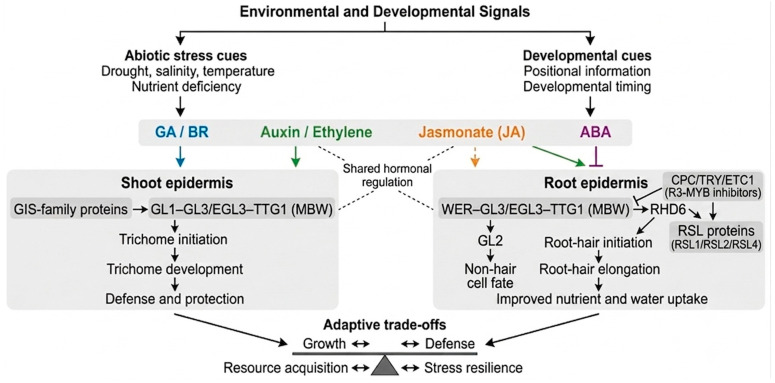
Core–Input–Output framework for environmentally responsive epidermal development. Environmental, nutritional, hormonal, and developmental inputs regulate organ-specific shoot and root networks. Arrowheads indicate activation or promotion, blunt-ended lines indicate repression or inhibition, and dashed lines indicate indirect, proposed, or context-dependent relationships. Colors distinguish regulatory components or signaling pathways.

Integrating current genetic, molecular, and hormonal evidence reveals that plant epidermal patterning is not a rigid developmental program but a dynamic regulatory network that is continuously modulated by environmental conditions [6,101,105]. This network is built upon conserved genetic pathways governing trichome and root-hair development, whose activities are coordinated by phytohormonal signaling and systemic regulatory hubs in response to developmental and abiotic cues. Together, these interconnected regulatory pathways shape epidermal plasticity by balancing the costs of epidermal structure formation with the benefits of stress resilience, resource acquisition, and environmental adaptation (Figure 11).

### 5.1. Core, Input, and Output Layers

The framework organizes environmentally responsive epidermal development into interconnected core, input, and output layers (Figure 11). In the shoot branch, GIS-family factors and the GL1–GL3/EGL3–TTG1 MBW complex promote trichome initiation. In the root branch, the WER–GL3/EGL3–TTG1 complex activates GL2 and specifies non-hair identity, whereas mobile R3-MYBs inhibit WER-based MBW activity and permit RHD6/RSL-dependent hair initiation and elongation [33,42]. Shared hormonal inputs connect these organ-specific pathways while preserving their distinct developmental outputs.

Environmental and hormonal inputs converge through both direct molecular interactions and coordinated pathway outputs. DELLA–BZR1/BES1 binding, EIN3/EIL1–RHD6 cooperation, JAZ–RHD6 repression, and PHR1–MYC2/JAZ regulation provide defined points of molecular integration [43,51,81,108]. Other combinations act in parallel or exert opposing effects on growth. These signals are translated into cell-fate specification, ROS–Ca^2+^-dependent polarized growth, cell wall remodeling, and changes in trichome or root-hair abundance. The Core–Input–Output framework links molecular regulation with organ-specific epidermal responses across developmental and environmental contexts.

Previous reviews have separately summarized C2H2 zinc-finger regulation of epidermal development, molecular mechanisms of trichome formation, and phytohormonal control of trichome development [6,101,105]. The present synthesis does not propose a universal MBW–GIS–RSL molecular module. Instead, its conceptual advance is to integrate these fields within an evidence-graded Core–Input–Output framework that (i) separates the GL1-based shoot MBW pathway from the WER-based root non-hair pathway; (ii) places GIS and RSL regulators on distinct organ-specific branches; and (iii) distinguishes developmental mechanisms, directly stress-tested responses, and stress-related extrapolations.

### 5.2. Adaptive Trade-Offs in Epidermal Patterning

Epidermal plasticity reflects trade-offs among growth, defense, and resource acquisition. Jasmonate-associated defense pathways and GA/BR-associated growth pathways jointly influence trichome investment, and enhanced secondary metabolism or defense can coincide with reduced vegetative growth [76,77]. At the signaling level, interactions among DELLA- and JAZ-regulated pathways offer a route for coordinating these competing demands [99].

A complementary trade-off involves resource exploration and conservation. Auxin and ethylene promote root-hair elongation and nutrient foraging, whereas ABA signaling under severe salinity suppresses RHD6 activity and hair growth [52,84]. The relative influence of these pathways changes with stress intensity, developmental stage, and genotype. Under phosphate limitation, the PHR1–EIN3/EIL1 module further coordinates organ-level resource allocation [118].

Systemic signals can also override local positional patterning. Under phosphate starvation, sub-epidermal ETC1 expression supplies a mobile pro-hair signal that promotes ectopic root-hair formation [61]. Together with the stress-responsive examples summarized in Table 6, these mechanisms illustrate how epidermal patterning is reconfigured to balance uptake, protection, and growth under changing environmental conditions.

## 6. Conclusions and Emerging Frontiers

Plant epidermal patterning emerges from interconnected genetic and hormonal regulatory networks that integrate developmental programs with environmental cues. Conserved regulators, including MYB–bHLH–WD40 (MBW) complexes, GIS-family C2H2 zinc-finger proteins, and ROOT HAIR DEFECTIVE SIX-LIKE (RSL) transcription factors, coordinate trichome and root-hair development and contribute to adaptive plasticity under changing environmental conditions. Phytohormone signaling provides a major integrative mechanism, while hubs such as DELLA proteins and PHR1 connect growth, defense, and nutrient status to epidermal differentiation. Together, these pathways enable plants to balance resource acquisition, protection, and growth in response to environmental challenges. Although conserved regulators provide promising targets, their native functions, pleiotropic costs, and field-level benefits must be validated in crop species before engineering applications can be claimed.

Future research should resolve the cell-type-specific regulatory landscapes that govern epidermal development and their dynamic responses to environmental stress. Single-cell and spatial transcriptomics, high-resolution imaging, and functional genomics will enable regulatory networks to be dissected at cellular resolution. Bridging the gap between model species and crops remains equally important: functional characterization of conserved regulators in agronomically important plants, combined with high-throughput phenotyping and genome-editing technologies, will facilitate the translation of fundamental discoveries into crop improvement. Comparative and evolutionary studies will further clarify how conserved pathways have diversified to generate the wide range of epidermal structures observed across plant species. Integrating multi-scale omics, evolutionary biology, and biotechnology will ultimately support the development of crops with improved resilience to environmental stress.

## Figures and Tables

**Table 1:** Genetic determinants of trichome initiation and morphogenesis.

Gene/Protein	Regulatory Tier/Complex	Species	Function in Trichome Development	Interacting Partners/Key Targets	References
GL1 (MYB), GL3/EGL3 (bHLH), TTG1 (WD40)	Core MBW Activator Complex	*A. thaliana*	Promotes trichome initiation and patterning. Forms the central transcriptional activator.	Binds to promoters of GL2, TRY; inhibited by R3 MYBs.	[16,28]
SlMYB72, SlbHLH150, SlTTG1	Core MBW Activator Complex	*S. lycopersicum*	Promotes multicellular trichome formation.	Represses SlCycB2; antagonized by SlMYB75.	[19]
CPC, TRY, TCL1	R3 MYB Inhibitors	*A. thaliana*	Mobile inhibitors; repress trichome fate in neighboring cells via competitive binding with R2R3-MYB to bHLH.	Compete with GL1 for GL3/EGL3 binding and inhibit MBW activity in neighboring epidermal cells.	[18,29]
SlTRY	R3 MYB Inhibitors	*S. lycopersicum*	Regulates trichome formation and root-hair development.	Functionally complements *A. thaliana cpc* mutant.	[29]
GIS, GIS2, ZFP5, ZFP6, ZFP8	C2H2 Zinc Finger Integrators	*A. thaliana*	Integrate GA and cytokinin signaling to control trichome initiation in inflorescence organs.	Act upstream of GL1 and GL3; ZFP5 directly targets ZFP8.	[17,30,31]
SDG26	Epigenetic & Post-transcriptional Regulators	*A. thaliana*	Histone methyltransferase; represses trichome initiation.	Increases H3K27me3 repressive marks on GL3, MYB23, ZFP5/6.	[23]
miR319/TCP module	Epigenetic & Post-transcriptional Regulators	*P. tomentosa*	Co-regulates trichome initiation with GA signaling.	TCP19 interacts with DELLA protein RGA to repress GL2 expression.	[32]

**Table 2:** Hormonal regulation of epidermal development and stress integration.

Key Regulators	Developmental Stage	Species	Function	Hormonal/Environmental Input	References
WER-GL3/EGL3-TTG1 (MBW) → GL2	Cell Fate Patterning	*A. thaliana*	Specifies non-hair cell fate in N-position cells.	High BR signaling inhibits BIN2, allowing WER–GL3/EGL3–TTG1 activity and GL2 expression. Active BIN2 under low BR phosphorylates EGL3/TTG1 and inhibits the complex.	[42,60]
CPC, ETC1 (R3 MYBs)	Cell Fate Patterning	*A. thaliana*	Promote hair cell fate in H-position cells; mobile inhibitors.	Pi starvation induces sub-epidermal ETC1 expression.	[61]
RHD6, RSL1 (bHLH)	Initiation & Elongation	*A. thaliana*	Master regulators of root-hair initiation.	Activated by auxin and ethylene; targeted by JAZ repressors.	[43,51]
RSL4 (bHLH)	Initiation & Elongation	*A. thaliana*	Master regulator of root-hair elongation; determines final hair length.	Directly activated by EIN3-RHD6 complex (ethylene), ARR1/12 (cytokinin), PHR1 (Pi stress).	[43,44,62]
GTL1, DF1 (Trihelix)	Negative Regulators	*A. thaliana*	Repress root-hair growth to determine final size.	Directly bind *RSL4* promoter to fine-tune elongation.	[48]
OBP4 (DOF TF), ABFs (bZIP TFs)	Negative Regulators	*A. thaliana*	Mediate ABA-induced suppression of root-hair growth.	OBP4 represses RSL2; ABFs interact with and suppress RHD6 activity.	[52,63]

**Table 3:** Phytohormonal regulation of trichome and root-hair development under environmental stress.

Hormone	Effect on Trichomes	Effect on Root Hairs	Key Signaling Components & Molecular Mechanisms	Regulatory Relationships and Stress Context	References
Gibberellin (GA)	Promotes initiation and branching.	Negative regulator of elongation.	Degrades DELLA repressors. DELLAs inhibit trichome activators and BZR1. Upregulates GL1 expression.	Direct DELLA–BZR1/BES1 binding connects GA and BR signaling. ABA often produces an opposing root-growth output under severe stress.	[81,82,84]
Brassinosteroid (BR)	Promotes initiation in some shoot contexts; provides a parallel positive input with GA-related pathways.	High BR promotes non-hair fate and reduces root-hair formation; active BIN2 under low BR favors hair fate.	High BR inhibits BIN2, allowing WER–GL3/EGL3–TTG1 activity and GL2 expression. Active BIN2 phosphorylates EGL3/TTG1 and inhibits the complex.	Direct DELLA–BZR1/BES1 interaction connects BR and GA. Root effects depend on BR control of BIN2.	[60,81,104]
Auxin	Promotes initiation.	Positive regulator of initiation and elongation.	Regulates RSL gene expression. Modulates polar auxin transport to root epidermis.	Converges with ethylene-regulated pathways; central to the low-Pi root-hair response.	[50,84,105]
Ethylene	Promotes initiation.	Positive regulator of initiation and elongation; induces ectopic hairs.	EIN3/EIL1 physically interact with RHD6/RSL1 to activate RSL4.	Converges with auxin-regulated pathways; contributes to cadmium-induced root-hair elongation.	[43,106,107]
Jasmonate (JA)	Promotes elongation (e.g., in tomato).	Positive regulator of elongation.	JAZ repressors interact with and inhibit RHD6/RSL1 and MYC2. Degradation of JAZs releases these TFs.	JAZ proteins also interact with PHR1, connecting JA and phosphate signaling; JA and ethylene can produce parallel positive root-hair outputs.	[51,75,108]
Abscisic Acid (ABA)	Context-dependent; can be inhibitory.	Negative regulator of elongation under severe stress.	Induces OBP4 (represses RSL2) and ABFs (inhibit RHD6 activity).	Often opposes auxin/ethylene-driven growth under severe stress and contributes to growth–defense allocation.	[52,63]
Cytokinin (CK)	Promotes initiation.	Positive regulator of elongation under specific stresses (e.g., Pi deficiency).	Type-B ARRs (ARR1, ARR12) directly upregulate RSL4 expression.	Coordinates with ethylene biosynthesis and acts downstream of phosphate sensing in specific contexts.	[62,95]

**Table 4:** Key regulators of ROS–Ca^2+^-dependent polar growth in root hairs.

Component	Gene/Protein	Function in Polar Growth	Regulation	Interdependence	References
ROS Production	RBOHC/RHD2 (NADPH Oxidase)	Primary source of apoplastic superoxide for root-hair elongation; establishes tip-high ROS gradient.	Activated by Ca^2+^, ROP GTPases, and phosphorylation. Directly upregulated by RSL4.	Its activity is required for Ca^2+^ influx. Creates a positive feedback loop.	[109,110,111]
	PRX44 (Class III Peroxidase)	Involved in ROS metabolism and cell wall modifications during root-hair initiation.	Transcriptionally upregulated by ethylene via EIN3/EIL1.	Cooperates with RBOHC in ethylene-induced root-hair formation.	[106]
ROS Homeostasis	Flavonols (e.g., via TT4)	Antioxidants that spatially restrict ROS accumulation.	Synthesized in specific epidermal cells.	Loss of flavonols (*tt4* mutant) leads to ectopic ROS and increased root-hair initiation.	[114]
Ca^2+^ Signaling	CNGCs (Cyclic Nucleotide-Gated Channels)	Mediates Ca^2+^ influx to establish a tip-focused cytosolic Ca^2+^ gradient and oscillations.	Activated by ROS and hyperpolarization.	Required for sustained RBOHC activity.	[111]
Signaling Hub	ROP2 (ROP GTPase)	Molecular switch for cell polarity; coordinates actin cytoskeleton and vesicle trafficking.	GTP-bound form activates RBOHC.	ROP2 activation requires functional RBOHC; forms a regulatory node.	[109]

**Table 6:** Representative studies of stress-responsive trichome and root-hair development.

Species/Tissue	Stress or Treatment	Regulator or Pathway	Epidermal Phenotype	Experimental Basis	References
*A. thaliana* root epidermis	Drought and salinity	PIN1-dependent auxin distribution	Cell-type-specific changes in epidermal development	Stress treatment with single-cell transcriptomic analysis	[9]
*A. thaliana* root epidermis	Phosphate starvation	AL6/ETC1; PHR1; EIN3/EIL1; ARR1/ARR12–RSL4	Ectopic root hairs and increased hair length	Nutrient-stress experiments with genetic and transcriptional analyses	[61,62,108,119]
*A. thaliana* roots	Boron deficiency	JA biosynthetic program and JAZ–RHD6/RSL regulation	Reduced primary-root growth with enhanced root-hair development	Low-boron treatment linked to JA biosynthesis and JAZ–RHD6/RSL signaling	[51,71,78]
*A. thaliana* root hairs	Salinity	ABA-activated ABFs interacting with RHD6	Inhibited root-hair elongation	Salinity treatment with genetic and protein-interaction analyses	[52]
*A. thaliana* root hairs	Cadmium	ROS accumulation and cell wall-related genes	Enhanced root-hair elongation	Cadmium treatment with physiological, pharmacological, and gene-expression analyses	[107]
*A. thaliana rhd2* and *der1* mutants	Drought	RHD2/ROS, actin cytoskeleton, and aquaporins	Genotype-specific drought responses	Mutant physiological analysis under drought	[15]
*Shepherdia × utahensis* leaves	Water availability	Trichome-density response	Altered trichome density and plant growth	Water-availability treatment with trichome and growth measurements	[123]
*Zea mays* root hairs	Cold stress	Cold-responsive morphological and transcriptomic programs	Intensity-dependent root-hair changes	Cold-stress morphology and transcriptomic analysis	[11]
*Triticum aestivum* roots	Drought and salinity	Root phenotype and gene-expression responses	Altered root traits under stress	Drought/salinity phenotyping and gene-expression analysis	[69]

## Data Availability

No new data were created or analyzed in this review. Data sharing is not applicable to this article.
